# Effects of cold plasma seed treatment on pea (*Pisum sativum* L.) plant performance under drought and well-watered conditions

**DOI:** 10.1371/journal.pone.0322108

**Published:** 2025-05-02

**Authors:** Dhanuja N. Abeysingha, Shifa Dinesh, Samitha Madushani Kottage, Lingyun Chen, M. S. Roopesh, Malinda S. Thilakarathna

**Affiliations:** Department of Agricultural, Food and Nutritional Science, University of Alberta, Edmonton, Canada; Universidade de Coimbra, PORTUGAL

## Abstract

Cold plasma (CP) technology is an emerging technology with the potential to enhance agricultural productivity and sustainability. Although its application in crop production is still in the early stages, CP seed treatment has demonstrated promise in improving various growth parameters, especially in legumes. We hypothesized that CP seed treatment can improve nodulation, symbiotic nitrogen fixation (SNF), root and shoot growth, overall productivity, and drought stress resistance in field pea. A controlled environmental study was conducted to investigate the effects of dielectric barrier discharge-generated CP seed treatment for 6 min on yellow field pea under different moisture regimes [30%, 45%, 60%, and 75% field capacity (FC)], focusing on nodulation, and root and shoot growth parameters at the flowering stage. Based on experiment-1 findings, 30% and 75% FC were selected as drought and well-watered conditions, respectively, to study the effect of CP seed treatments on SNF parameters at the flowering stage and nitrogen fixation, yield, and seed quality parameters at maturity. CP seed treatment improved root growth parameters at the flowering stage and an increasing trend was observed for shoot and nodulation parameters across different moisture levels. As an independent factor, moisture stress negatively affected nodulation and shoot growth parameters at the flowering stage. CP seed treatment improved nitrogen fixation and yield parameters under well-watered conditions compared to drought conditions at seed maturity. However, the seed protein content or the quality was not improved by the CP seed treatment. Grain yield, yield parameters, grain nitrogen, and nitrogen fixation were reduced under drought stress compared to the well-watered condition. Therefore, these findings underscore the potential of CP to enhance crop performance in well-watered conditions. The underperformance of the CP-treated seeds at drought conditions is not well understood and warrants further investigation.

## Introduction

Limited nitrogen (N) availability poses a challenge to global protein production. However, advances in biological and chemical N fixation have significantly enhanced our ability to meet the growing protein demands of an expanding global population [1]. Legumes, including pulses, fix atmospheric N_2_ into ammonia through symbiosis with rhizobia bacteria [1,2]. This biological nitrogen fixation benefit legumes and neighboring or following non-legume crops while reducing the reliance on inorganic N fertilizers and supporting sustainable agriculture [2]. Additionally, pulses such as peas contribute to crop production and disease management through intercropping and crop rotation [3,4]. They offer an affordable, nutritious, and environmentally friendly protein source with a lower carbon and water footprint than meat and dairy products [5].

Despite their advantages, pulse production is expected to be highly affected by future climate conditions, which will be marked by more frequent extreme weather events [6]. These abiotic stress conditions, including heat, drought, and salinity, increase variability and vulnerability in crop yields, posing a substantial threat to food security [6]. Therefore, while pulses offer a sustainable protein alternative, it is crucial to address their limitations and the broader impacts of climate change. Drought is one of the major environmental factors that negatively affects pulse production. Drought is characterized by a prolonged deficiency in water supply, which can manifest in various forms: atmospheric (below-average rainfall), agricultural (insufficient irrigation supply), soil (reduced surface and groundwater levels), and physiological (transpiration exceeding water uptake) [7]. Pulse crops, including field pea, can experience both short-term and long-term drought stresses. Drought leads to reduction in carbon assimilation, stomatal closure, osmotic adjustment, growth inhibition, hydraulic changes, cellular drought signaling, repressed shoot growth, seed and pod abortion, and yield losses in pea [8–11]. Additionally, drought can hinder root nodulation and N fixation in pulses, resulting in reduced protein production [12]. Drought tolerance in pea can be managed by adopting strategies such as varietal screening, breeding, and marker-assisted selection [11]. However, these methods are time consuming and can take many years to develop cultivars that are tolerant to drought. Therefore, it is urgently necessary to explore alternative methods for enhancing drought tolerance in peas, especially in light of current climate change scenario and the increasing demand for food security due to population growth.

Cold plasma (CP) seed treatment is a rapid, cost-effective, and environmentally friendly method to enhance seed performance, plant growth, and overall plant productivity [13–15]. As the fourth state of matter, plasma consists of excited atomic, molecular, ionic, and reactive species, including electrons, ions, free radicals, and gas molecules [16]. Reactive oxygen and nitrogen species (RONS) are particularly influential in CP, affecting plant growth and development. In low concentrations, these reactive species may act as signaling molecules like plant hormones, regulating growth, development, metabolism, and stress responses [16, 17]. The CP seed treatment can significantly influence various aspects of plant development and physiological processes in legumes, such as promoting seed germination and seedling growth, activating photosynthesis, regulating carbon and N metabolism, and facilitating symbiotic nitrogen fixation (SNF) [14–16,18–22]. CP seed treatments have the potential to influence the physical properties of seeds and affect gene expression and may have a lasting impact on the nodulation, SNF, growth, and yield of legumes. Although, previous research has demonstrated the benefits of CP on legume crops, its effects on pulses under drought stress remain unexplored. The objective of this study was to explore the effects of CP treatment on pea plant performance under different moisture conditions such as drought and well-watered conditions. We hypothesized that seed treatment with dielectric barrier discharge (DBD) atmospheric cold plasma can improve nodulation, SNF, root and shoot growth, overall productivity, and drought stress resistance in field pea. We anticipate this study will provide new insights into optimizing pea cultivation under varying water regimes to meet the increasing protein demand of a growing population in the context of climate change.

## Materials and methods

### CP seed treatment

All the experiments reported in this study were conducted under greenhouse conditions at the University of Alberta, Canada. The yellow pea cultivar CDC Meadow [23] was selected for this study as it is the most widely grown pea cultivar in the province of Alberta, Canada. In each experiment, seeds of the CDC Meadow were subjected to CP treatment using the following process. Seeds were exposed to CP for 6 min using a highly precise DBD system (Advanced Plasma Solutions in Malvern, PA, USA), following the procedure outlined in Abeysingha et al. (2024) [18]. In brief, the DBD system consisted of a high-voltage electrode connected to a high-voltage generator (voltage: 0–34 kV, power: approximately 300 W) and operated at specific parameters: an output frequency of 3.5 kHz, a duty cycle of 70%, and an output pulse width of 10 μs. The treatment distance between the plasma source and the pea seeds was consistently set at 2–3 mm, while the distance separating the electrodes was maintained at 4–5 mm.

### Experiment 1: Assessing the CP seed treatment effects on plant growth and nodulation in pea under four different field capacity levels

#### Plant materials and growing conditions.

Experiment 1 was conducted with the objective of investigating the effects of the CP treatment on pea plant growth under four different moisture conditions. CP-treated and untreated control seed samples were directly planted in 3.8 L pots. Each pot was filled with 1600 g of a growing medium composed of a 2:1 (v:v) mixture of Sunshine #4 potting mix (Sun Gro Horticulture, Vancouver, Canada) and sand (Quikrete premium play sand, Target products, Canada). This mixture was thoroughly blended using a soil mixer for approximately 10 min to ensure uniformity. Each pot was lined with low-density polyethylene (LDPE) pot liner before adding the soil to prevent water from leaching and to retain the growing medium. Seeds in pots were inoculated with 1 ml of *Rhizobium leguminosarum* biovar *viciae* 3841, a compatible rhizobia strain for field pea, where rhizobial density was adjusted to OD_600_ = 0.1 [24]. Initially, two seeds were sown in each pot, then thinned to one seedling per pot approximately one week after germinating.

Before initiating the experiment, the field capacity (FC) of the potting mix was determined following the method outlined by Liyanage et al. (2022) [25]. Plants were exposed to four soil moisture levels (30% FC, 45% FC, 60% FC, and 75% FC) by adjusting FC levels of growing media immediately after planting until the plants were harvested. Each pot was watered daily to maintain the desired FC, and the moisture level of the pots was adjusted gravimetrically. The experiment was structured as a complete randomized design with eight treatments [4 FC levels (30% FC, 45% FC, 60% FC, and 75% FC) × two seed treatments (CP and control)]. Each treatment consisted of 8 pots (*n* = 8) planted randomly within each FC level. Each pot was fertilized twice weekly with 50 ml of quarter-strength N-free Hoagland’s nutrient solution (Caisson Labs, UT, USA). All the plants were grown under greenhouse conditions [24 ± 4°C light/16 ± 4°C dark, with a 16/8 h light/dark photoperiod and an average supplemental light intensity of 500 ± 100 μmol m^2^s^-1^ (Philips Silhouette high output fluorescent bulbs F54T5/835 Holland, Alto Collection)].

#### Data collection.

Plants were harvested at the 50% flowering stage (BBCH 65), and their roots were carefully washed to remove the planting medium. Any remaining particles attached to the roots were manually removed with tweezers. Subsequently, nodules from each plant were manually extracted and counted. The roots were scanned using an Epson Expression 1640 scanner (Epson Canada Ltd., Canada), and detailed root morphological analyses were conducted using WinRHIZO software (Regent Instruments Inc., Canada), including measurements of total root length, root volume, and root surface area. Dry weights of nodules, shoots, and roots were determined after drying the plant materials in a hot air oven at 60°C for three days.

### Experiment 2: Assessing the CP seed treatment effects on plant growth, nodulation, SNF, and yield in pea under drought and well-watered conditions

#### Plant materials and growing conditions.

Experiment 2 aimed to evaluate the specific effects of CP seed treatment on SNF-related parameters during the flowering stage, as well as on yield-related parameters and nitrogen fixation at maturity, under well-watered (75% field capacity) and drought (30% field capacity) stress conditions as mentioned below. Based on the results from Experiment 1, 30% FC was selected as the drought treatment for this experiment. In this experiment, the plant growing medium and pot preparation, rhizobia inoculation, nutrient application, CP seed treatment application, seeding, maintaining the desired FC level, and maintaining the greenhouse conditions were the same as in Experiment 1. The yellow pea cultivar CDC Meadow was used, and the study was structured as a completely randomized design with four treatments: two FC levels (30% FC and 75% FC) × two seed treatments (CP-treated and control). Each treatment consisted of 20 plants, planted randomly within each FC level. Additionally, a non-nodulating control [Frisson P56 (nod-) (John Innes Institute, Norwich, UK)] plants were grown (*n* = 20) under 75% FC using the same growing media along with the N-fixing plants and used as the reference plant for calculating SNF. Each pot was fertilized twice weekly with 50 ml of quarter-strength N-free Hoagland’s nutrient solution. Plants were labeled with 50 mL of 0.5 mM K^15^NO_3_ solution (10 atom% ^15^N; 348481-25G; Sigma Aldrich, Oakville, ON, Canada) three times every other week starting from two weeks after germinating to measure the SNF capacity.

#### Data collection.

**Evaluation of plant growth, nodulation, and SNF at the flowering stage:** In this experiment, half of the plants in each treatment group (*n* =10) were harvested at the 50% flowering stage (BBCH 65). Before harvest, leaf chlorophyll content (SPAD) and linear electron flow (LEF) were measured on fully expanded young leaves using a MultispeQ handheld unit (PHOTOSYNQ Inc. MI, USA) on plants at the 50% flowering stage. Three readings were taken per plant, each from distinct leaves of similar maturity. Subsequently, plants were harvested, their roots were carefully washed, nodules were counted and collected, and the root scanning and measurement of root morphological parameters were conducted following the procedures outlined in Experiment 1. Dry weights of nodules, shoots, and roots were determined after drying the plant materials in a hot air oven at 60°C for three days.

**Evaluation of yield parameters:** The other half of the plants in each treatment group (*n* = 10) were maintained until maturity and were harvested at the BBCH 89 growth stage (fully ripe: all pods dry and brown, seeds dry and hard). Pods and shoots were then dried in a hot air oven at 60°C for three days, and data on pod number, pod weight, seed number, and seed weight were recorded.

**Evaluation of tissue total N, carbon isotope discrimination (CID), and symbiotic N fixation:** Oven-dried tissue samples (shoot and seed) from Experiment 2 were separately ground to a medium-fine consistency using a coffee grinder. A sub-sample of each ground sample was further ground using a bead beater homogenizer (OMNI International, Kennesaw, GA, USA) to produce an ultra-fine homogenized powder. Ground tissue samples (5 mg from each replicate in each treatment) were encapsulated into tin capsules (8 mm × 5 mm, D1008, Isomass Scientific Inc., Calgary, AB, Canada). These tin capsules were arranged in a 96-well plate and sent to the Stable Isotope Facility at Agriculture and Agri-Food Canada’s Lethbridge Research and Development Centre for ^15^N, ^13^C, total N%, and total C% analysis. The samples were analyzed using a Finnigan Delta V plus Isotope Ratio Mass Spectrometer (IRMS) equipped with a Flash 2000 Elemental Analyzer (Thermo Fisher Scientific, Voltaweg, The Netherlands) and a Conflo IV (Thermo Fisher Scientific, Bremen, Germany) interface between the IRMS and the analyzer. Then, the deviation between the isotopic composition of the PeeDee Belemnite (PDB, a calcareous fossil) standard and the carbon isotope of the samples was determined according to a method described by Mutanda et al. (2024) [26].

The samples were analyzed using the following equation:


$$\text{δ}\left[\text{‰}\right]=\frac{\text{Rp}-\text{Rs}}{\text{Rs}}$$ (1)

where, Rp is the isotopic abundance in the pea tissue sample, and Rs is the abundance ratio ^13^C/^12^C of the standard, for which a fossil from the Pee Dee Formation (Pee Dee Belemnite, PDB) was used. The carbon isotope discrimination (CID) was calculated as follows:


$$\text{CID}\left[\text{‰}\right]=\frac{\text{δa}-\text{δp}}{1+\text{δp}}\times 1000$$ (2)

Where, δa is the isotopic composition of the atmosphere (approximately −8‰) and δp is the isotopic composition of the shoot or seed sample [26].

The percentage of N derived from the atmosphere (%Ndfa) in pea shoots and seeds was calculated using the isotope dilution technique using the following formula [27].


$$\%\text{Ndfa}=\left(1-\frac{\text{atom}{\%}^{15}\text{N }{\text{excess}}_{\text{N fixing pea}}}{{\text{atom\% }}^{15}\text{N }{\text{excess}}_{\left(\text{N non-fixing pea}\right)}}\right)\times 100$$ (3)

where atom% ^15^N excess is the atom% ^15^N in the pea in excess of natural abundance level (0.3663). It was calculated as follows:


$${\text{atom\% }}^{\text{15}}{\text{N excess}}_{\text{(pea)}}\;={\text{ atom\% }}^{\text{15}}{\text{N}}_{\text{(pea)}}-\text{0.3663}$$ (4)

The fixed N amount in the shoot and seeds was determined based on the total aboveground N content and %Ndfa as follows:


$$\text{Total N fixed }=\text{total tissue N content of peas}\times \frac{\text{\%Ndfa}}{100}$$ (5)

**Pea protein isolation and protein content analysis:** Due to the insufficient availability of samples from drought-stressed plants, and the necessity of having an adequate sample size to obtain reliable and accurate results, we only conducted protein analysis on seeds harvested from well-watered plants, including both CP-treated and untreated controls.

***Pea protein isolation:*** Four ground seed samples from CP-treated and untreated plants subjected to the 75% FC treatment were sent to the Plant Protein Lab, University of Alberta, for total protein content and Sodium dodecyl-sulfate polyacrylamide gel electrophoresis (SDS-page) analyses [28]. Pea flour was defatted using a double hexane extraction with a flour-to-hexane ratio of 1:5 for 2 hr each. The pea flour was separated from hexane by centrifugation at 8,000 g for 15 min. Then, the pea flour was air-dried in a fume hood at 22°C overnight.

***Alkaline extraction followed by isoelectric precipitation (AI):*** Defatted pea flour was dispersed in distilled water at flour to the water of 1:10. Then the pH of the mixture was adjusted to 9 using 1 mol/l NaOH and stirred for 2 hr at room temperature (~22°C). The suspension was centrifuged at 8000× g for 15 min at 4°C. The supernatant was adjusted to pH 4.5 using 1 mol/l HCl to precipitate the proteins, which were then collected by centrifugation at 8000 g for 15 min at 4°C. The pea protein samples were subjected to alkaline extraction followed by isoelectric precipitation and neutralization by 1 mol/l NaOH before freeze drying [28].

***Protein content analysis:*** After freeze-drying, pea protein samples were stored in plastic containers at 4°C. The protein content was determined by a Leco N analyzer (FP-428, Leco Corporation, St. Joseph, MI, USA) using a protein conversion factor of 6.25 [29].

***Sodium dodecyl-sulfate polyacrylamide gel electrophoresis (SDS-page) analysis:*** The SDS-Page analysis was carried out to study the protein profiles of CP-treated and untreated samples at 75% FC level under non-reducing conditions. The extracted protein samples (3 mg/mL, protein-based) were mixed with the buffer solution, which contains 0.125 M Tris–HCl at pH 6.8, 4% w/v SDS, 20% v/v glycerol, and 1% bromophenol blue w/v. Then, the mixed sample solutions were heated at 100°C for 5 min, followed by cooling down to room temperature (23°C). The samples were centrifuged at 3500 x g for 5 min and the supernatant was used for the study. Precision Plus Protein™ Standard #161–0374 (Bio-Rad Lab., Hercules, CA, USA) was used as the standard marker for the analysis, and it was loaded with 5 μL in a separate lane on 4% stacking gel and 12% separating gel plate. The sample supernatants were added with 12 μL per well of the SDS-Page gel. A voltage of 80 V was maintained throughout the electrophoresis process. After electrophoresis, the gel was stained with 0.1% (w/v) Coomassie Brilliant Blue R-250 for 25 min followed by the de-staining with water, methanol, and acetic acid at a ratio of 4:5:1 (v/v/v) overnight [30].

### Statistical analyses

In both Experiments 1 and 2, normality and homogeneity of variance for each data parameter were assessed using the Shapiro-Wilk and Levene’s tests, respectively. In experiment 1, data for each parameter were analyzed using a two-way Analysis of Variance (ANOVA) to assess the main effects of FC level and seed treatment and their interaction (FC level × seed treatment). Despite the insignificant interaction effect, we conducted a targeted single degree of freedom contrast analysis comparing the control and CP seed treatments at each FC level. This analysis aimed to uncover any treatment effects of potential agronomic importance. In Experiment 2, data associated with each parameter collected at flowering or maturity stages were analyzed using a two-way ANOVA, similar to Experiment 1. In cases where no significant interaction effect was observed, only comparisons relating to the main effects were addressed in the results section. However, for datasets exhibiting a significant interaction effect, mean separations were conducted for the interactions between FC levels and seed treatments using Tukey’s procedure, with a type I error maintained at 0.05 (SAS Institute Inc., Cary, NC, USA, 2010). Protein content data were analyzed using a two-sample T-test. Statistical significance for all the analyses were declared at P ≤ 0.05.

## Results

### Experiment 1: Assessing the CP seed treatment effects on plant growth and nodulation in pea under four different field capacity levels

#### Effect of CP seed treatment and FC levels on nodule parameters.

Compared to the 75% FC level, the number of nodules decreased by 33% and 69% and the nodule dry weight decreased by 44% and 58% for the 45% and 30% FC levels, respectively (Table 1). While the main effect of CP seed treatment was not statistically significantly for the evaluated nodule traits, the CP treatment did show an increasing trend in nodule number (14% increase) and nodule dry weight (8% increase) compared to the control (Table 1). This was an indication that the CP seed treatment has a potential to enhance nodule number and dry weight in pea plants. Similarly, at each FC level, nodule number showed an increasing trend for CP seed treatments, with increases of 10%, 23%, and 20% observed at the 75% FC, 60% FC, and 45% FC levels compared to the untreated control (Table 1). Nodule dry weight also showed an increasing trend for the CP treated, with 5%, 10%, 4%, and 25% increments observed at the 75% FC, 60% FC, 45% FC, and 30% FC levels compared to the untreated control (Table 1). Overall results indicated that moisture stress negatively affected nodulation parameters, whereas CP seed treatment tended to improve these parameters under both well-watered and moisture-deficient conditions.

**Table 1:** The effect of cold plasma seed treatment and soil moisture levels on nodulation, root, and shoot parameters of pea plants at the flowering stage. All parameters were indicated as per plant.

Treatments			Nodule parameters		Root and shoot parameters				
**Main and interaction effects**	**Seed treatment** ^**⁑**^	**FC** ^**‡**^	**Nodule dry weight (mg)**	**Nodule number**	**Root dry weight (mg)**	**Root length (cm)**	**Root surface area (cm**^**2**^)	**Root volume (cm**^**3**^)	**Shoot dry weight (mg)**
	**Control**	**75%**	94 a^†^	289 a	248 a	3195 a	393 a	3.9 a	5413 a
	**CP**	**75%**	99 a	319 a	301 a	3771 a	523 a	5.8 a	5765 a
	**Control**	**60%**	69 a	243 a	268 a	3488 a	404 a	4.1 a	5539 a
	**CP**	**60%**	76 a	299 a	333 a	3656 a	526 a	6.0 a	5259 a
	**Control**	**45%**	53 a	186 a	277 a	3477 a	441 a	4.2 a	4984 a
	**CP**	**45%**	55 a	223 a	259 a	3523 a	475 a	4.8 a	4668 a
	**Control**	**30%**	36 a	90 a	190 a	2873 a	378 a	4.0 a	3249 a
	**CP**	**30%**	45 a	87 a	232 a	3271 a	434 a	4.6 a	3588 a
**Seed treatment x FC**			**NS** ^*^	**NS**	**NS**	**NS**	**NS**	**NS**	**NS**
	**Non-CP**		63 m	204 m	246 n	3258 n	404 n	4.0 n	4796 m
	**CP**		68 m	232 m	281 m	3556 m	489 m	5.3 m	4820 m
**Seed treatment**			**NS**	**NS**	**S**	**S**	**S**	**S**	**NS**
		**75%**	96 p	304 p	274 p	3483 p	458 p	4.9 p	5589 p
		**60%**	72 pq	271 pq	301 p	3572 p	465 p	5.1 p	5399 p
		**45%**	54 qr	205 q	268 p	3500 p	458 p	4.5 p	4826 q
		**30%**	40 r	93 r	211 q	3072 p	406 p	4.3 p	3418 r
**FC**			**S**	**S**	**S**	**NS**	**NS**	**NS**	**S**

⁑ Cold plasma (CP) seed treatment was given to pea seeds for 6 mins using a DBD cold plasma generating system. Values in the table are expressed as the mean (n = 10).

‡ FC = Field capacity, pots were maintained at four FC levels 75%, 60%, 45%, and 30%.

* NS = non-significant, S = significant.

means followed by the same letter indicate means are significantly similar within each FC level (a), within each parameter by the single degree of freedom contrast analysis, P ≤ 0.05. Means followed by the same letter are not significantly different among CP treatment main effect means (m, n), and among FC main effect means (p-r) within each parameter by the LSD test, P ≤ 0.05.

#### Effect of CP seed treatment and FC levels on root and shoot parameters.

The lowest root dry weight was observed at the 30% FC level where, a 23% reduction was observed compared to the 75% FC level. Root dry weights were similar for the other FC levels (Table 1). No significant differences in root length, surface area, or volume were detected across the different moisture regimes. However, a decreasing trend in all these parameters was observed at the 30% compared to the 75% FC level. CP seed treatment enhanced all measured root parameters according to the seed treatments’ main effect analysis (Table 1). Plants derived from CP-treated seeds showed significant increases in root dry weight (14%), length (9%), surface area (21%), and volume (33%) compared to the control. However, in each FC level, the measured root parameters showed no significant difference between CP seed treatment and the control (Table 1) but showed positive increasing trends for root length (up to 18%), root surface area (7–33%), and root volume (14–48%) compared to the control. Shoot dry weight was reduced by 64% and 16% at 30% FC and 45% FC, respectively, compared to the 75% FC level (Table 1), indicating substantial reduction at lower moisture levels. According to the main effects analysis for seed treatment and the contrast analysis between control and CP seed-treated plants within each FC level, CP seed treatment had no significant impact on shoot dry weight (Table 1). Overall results indicated that moisture stress negatively affected shoot and root dry weight, whereas CP seed treatment generally enhanced root phenotypic parameters.

### Experiment 2: Effect of CP seed treatment on plant growth, N fixation, and yield of pea under drought (30% FC) and well-watered (75% FC) conditions

In Experiment 1, most of the measured parameters were significantly reduced under 30% FC compared to 75% FC. Although not statistically significant, there was an increase of over 10% in every measured parameter under 30% FC with CP seed treatment except for the nodule number. CP seed treatment showed an increase in measured parameters ranging from 5% to 49% under 75% FC compared to untreated plants. Altogether, findings from Experiment 1 indicated that CP seed treatment had a potential to enhance different pea plant traits under well-watered and drought-stress conditions. Therefore, to extend the investigation, we examined root, nodule, shoot, and SNF-related parameters at the flowering stage and yield-related parameters (pod number, pod weight, seed number, seed weight, shoot dry weight) and N fixation at the maturity stage under well-watered (75% FC) and drought (30% FC) stress conditions in Experiment 2.

### Effect of CP seed treatment on plant growth and SNF of pea plants at the flowering stage under drought and well-watered conditions

#### Effect of CP seed treatment and FC level on nodule parameters:

**Fig 1:**
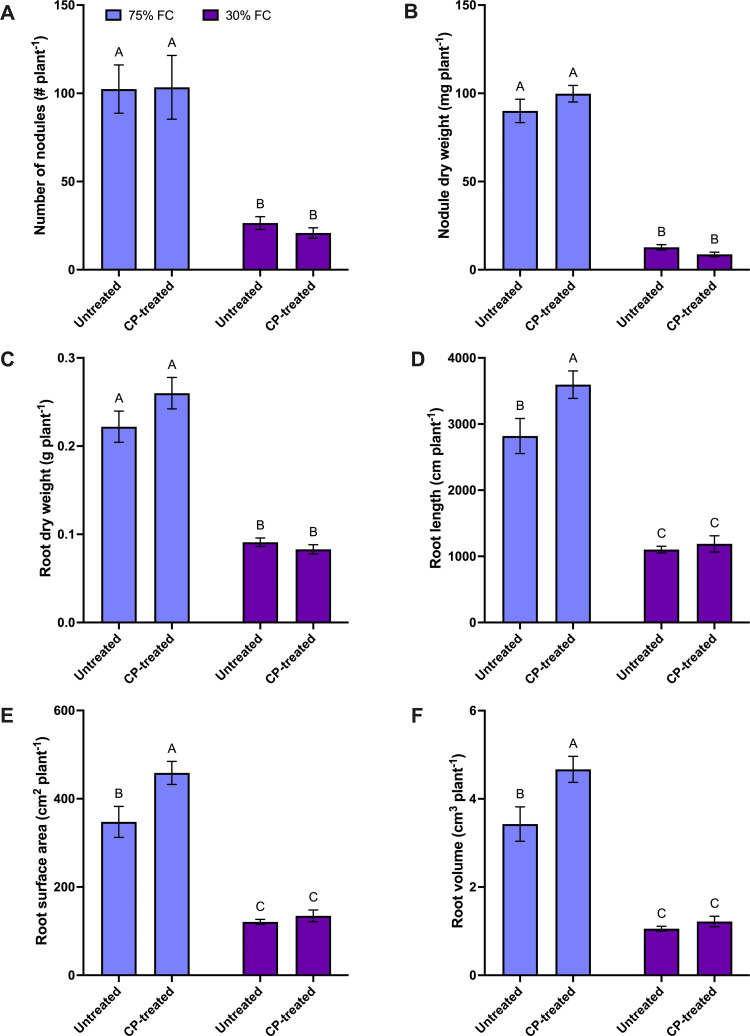
The effect of cold plasma (CP) seed treatment on nodulation and root growth parameters of pea plants at the flowering stage (BBCH 65) under 75% and 30% field capacity (FC) levels. (A) number of nodules per plant, (B) nodule dry weight (mg per plant), (C) root dry weight (g per plant), (D) root length (cm per plant), (E) root surface area (cm^2^ per plant), and (F) root volume (cm^3^ per plant). For all graphs, the error bars represent the SEM (n = 10). Bars followed by the same letter are not significantly different by the Tukey’s test, P ≤ 0.05.

There was a 77% reduction in total nodule number, an 89% reduction in total nodule dry weight, and a 59% reduction in average nodule dry weight at the 30% FC level compared to the 75% FC level (S1 Table). The CP seed treatment did not significantly influence nodule parameters (Figs 1A and 1B and S1 Table). However, both total and average nodule dry weight showed upward trends in the seed treatment main effect means (S1 Table). In particular, there was a 6% increase in total nodule dry weight and a 12% increase in average nodule dry weight in CP seed-treated plants compared to the control (S1 Table). Overall results indicated that moisture stress negatively affected nodulation parameters, while CP seed treatment showed no significant impact on these parameters.

**Effect of CP seed treatment and FC level on root parameters:** According to the FC main effect, root dry weight (62%), root length (64%), root surface area (68%), and root volume (73%) were considerably reduced at the 30% FC level compared to those observed at the 75% FC level (S1 Table). The CP seed treatment did not significantly increase root dry weight (S1 Table). However, compared to the untreated control, plants derived from CP-treated seeds had higher root length (18%), root surface area (27%), and root volume (32%) in the CP seed treatment main effect means (S1 Table). When considering the CP and FC treatment interactions, there was no difference in the measured root parameters of plants derived from CP-treated and untreated seeds under drought stress conditions (30% FC) (Figs 1C, 1D, 1E, and 1F). However, plants derived from CP-treated seeds exhibited a 32% increase in root surface area and a 38% increase in root volume at the 75% FC level (Figs 1E and 1F and S1 Table). Overall results indicated that moisture stress negatively affected root growth parameters, whereas CP seed treatment tended to improve these parameters only at higher moisture levels.

**Fig 2:**
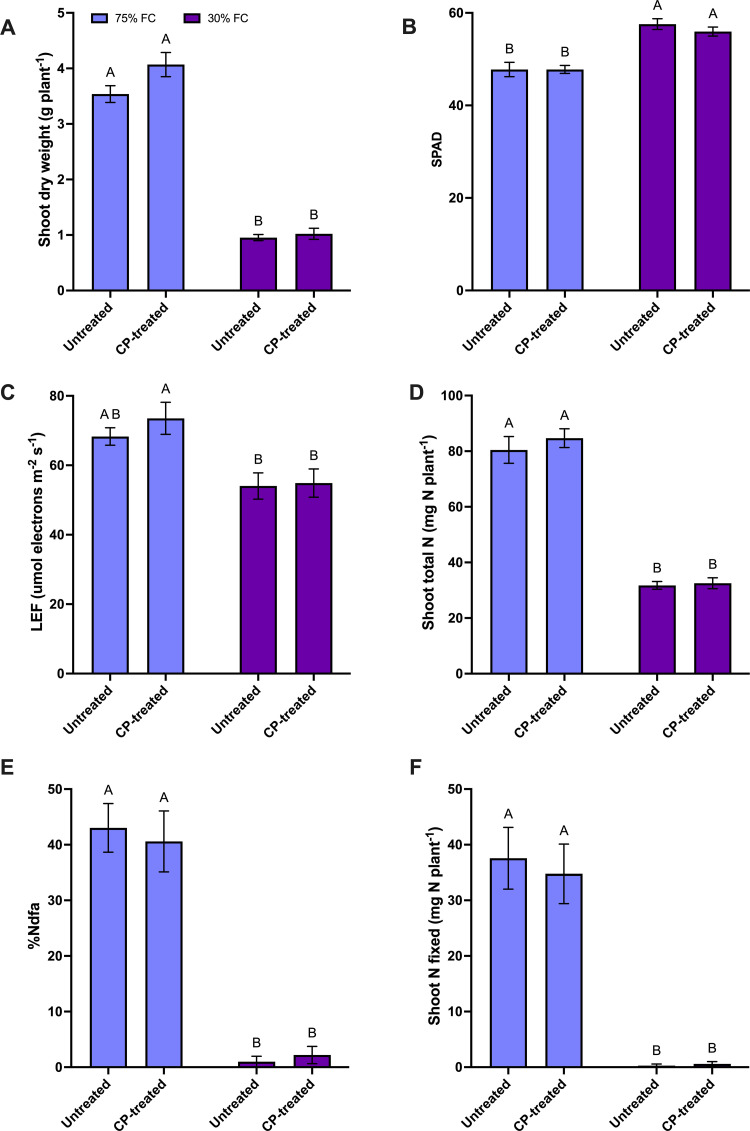
The effect of cold plasma (CP) seed treatment on root dry weight, photosynthetic parameters, and N fixation-related parameters of pea plants at the flowering stage (BBCH 65) under 30% and 75% field capacity (FC) levels. (a) shoot dry weight (g per plant), (b) SPAD, **(c)** LEF (µmol electrons m^-2^s^-1^ (d) shoot total N (mg N per plant) (e) percentage of N derived from the atmosphere (% Ndfa), and (f) total N fixed in shoot (mg N per plant). For all graphs, the error bars represent the SEM (n = 10). Bars followed by the same letter are not significantly different by the Tukey’s test, P ≤ 0.05.

**Effect of CP seed treatment and FC level on shoot dry weight, LEF, and SPAD:** According to the main effect of FC, drought stress (30% FC) lead to a significant decrease in shoot dry weight, with a reduction of 74% compared to the 75% FC level (S1 Table). Additionally, the LEF of drought-stressed plants was 24% lower than those grown at the 75% FC level (S1 Table). In contrast, relative leaf chlorophyll content (SPAD) showed a 19% increase in drought-stressed plants compared to the 75% FC level (S1 Table). On the other hand, plants from CP-treated seeds demonstrated a 12% increase in shoot dry weight compared to plants from untreated seeds (S1 Table). However, the CP seed treatment did not affect the LEF and SPAD (S1 Table). CP and FC interaction was not significantly different for shoot dry weight and LEF. However, increasing trends were observed for the aforementioned shoot parameters for CP seed treated plants compared to the untreated under 75% FC level (Fig 2). Overall results indicated that moisture stress negatively affected shoot growth parameters, whereas CP seed treatment tended to improve shoot growth regardless of the FC level.

**Effect of CP seed treatment and FC level on total shoot N content, N fixation-related parameters, and CID:** At the 30% FC level, total shoot N decreased by 61%, %Ndfa by 97%, total fixed N in shoots by 99%, and CID by 13%, compared to the 75% FC level (S2 Table). However, CP seed treatment did not affect any of those measured parameters (Fig 2).

### Effect of CP seed treatment on yield parameters and SNF of pea plants at maturity stage under drought and well-watered conditions

#### Effect of CP seed treatment and FC level on yield parameters.

**Fig 3:**
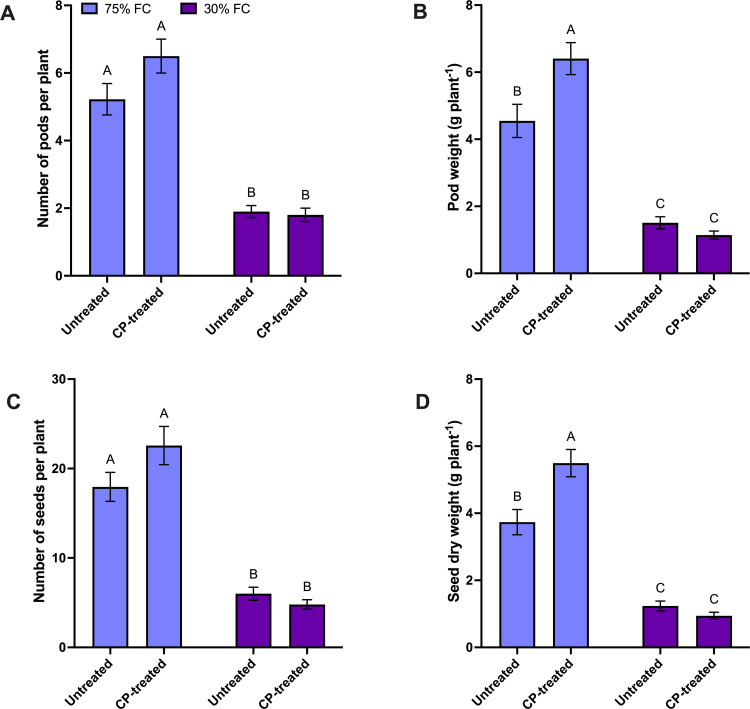
The effect of cold plasma (CP) seed treatment on yield parameters of pea plants at the maturity stage (BBCH 65) under 30% and 75% field capacity (FC) levels. (a) number of pods per plant, (b) pod weight per plant (g), (c) number of seeds per plant (d), (e) seed dry weight per plant (g). For all graphs, the error bars represent the SEM (n=10). Bars followed by the same letter are not significantly different by the Tukey’s test, P ≤ 0.05.

When considering the FC main effect, there was a 68% reduction in pod number, a 76% reduction in pod weight, a 73% reduction in seed number, and a 76% reduction in seed weight under 30% FC level compared to plants grown at the 75% FC level (S3 Table). According to CP seed treatment main effect analysis, the CP treatment did not impact pod number however, showed 25% higher pod weight, 14% higher seed number, and 30% higher seed weight than untreated plants (S3 Table). The interaction between the CP treatment and FC level was significant. Under drought stress, data for yield parameters were similar between the CP treatment and control whereas, under well-watered condition (75% FC), the yield parameters increased for CP treated (Fig 3). There was a 41% increase in pod weight, a 26% increase in seed number, and a 47% increase in seed weight in CP seed-treated plants compared to control plants under 75% FC (S3 Table). Overall results indicated that moisture stress negatively affected yield and yield quality parameters. The CP seed treatment tended to improve these yield parameters under well-watered conditions.

#### Effect of CP seed treatment and FC level on seed total N, N fixation related parameters, and CID.

**Fig 4:**
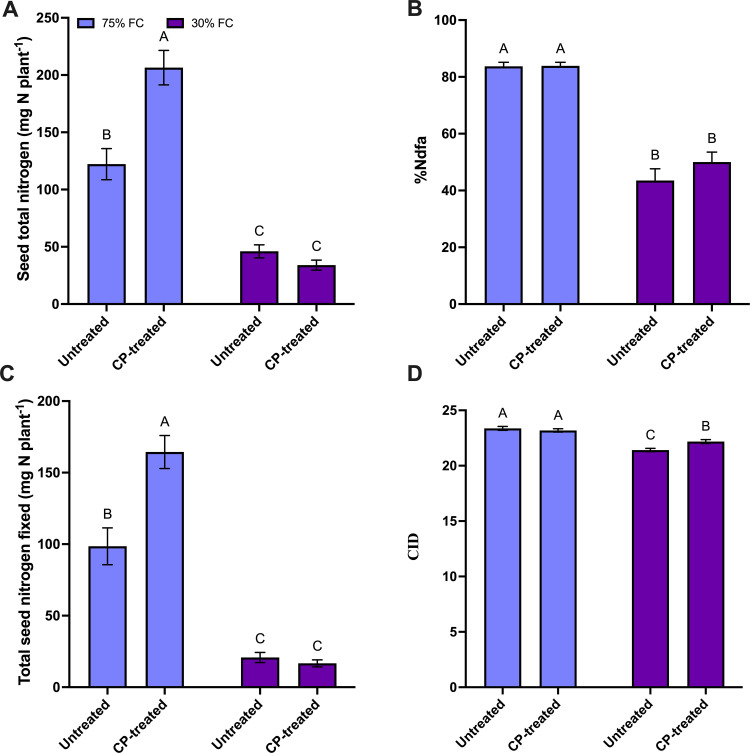
The effect of cold plasma (CP) seed treatment on N fixation-related parameters in seeds at the maturity stage (BBCH 65) under 30% and 75% field capacity (FC) levels. (a) seed total N (mg N per plant) (b) percentage of N derived from the atmosphere (% Ndfa), and (c) total N fixed in seeds (mg N per plant), (d) carbon isotope discrimination (‰). For all graphs, the error bars represent the SEM (n=10). Bars followed by the same letter are not significantly different by the Tukey’s test, P ≤ 0.05.

When considering the interactions between CP and FC treatments, significant differences were observed for both seed total N and total fixed N. Although there was no significant difference in seed total N and total fixed N between treated and untreated CP seeds at 30% FC, seed total N increased by 69%, and total fixed N in seeds increased by 66% for CP-treated plants compared to control plants at the 75% FC level (Figs 4A and 4C). There was no significant interaction between FC level and CP treatment for %Ndfa. The main effects of FC indicated a 46% decrease in %Ndfa in 30% FC plants compared to 75% FC plants (S3 Table) which was significant. The main effect of CP seed treatment indicated no significant difference between CP-treated and untreated plants for %Ndfa however, the CP seed treatment trended towards a 6% increase compared to control plants (S3 Table). When considering the CID data, there was no difference at the 75% FC level for CP treated seeds however, at the 30% FC level, CID was reduced in CP-treated plants compared to control plants (S3 Table). Overall results indicated that moisture stress negatively affected nitrogen fixation parameters, whereas CP seed treatment tended to improve these parameters, especially under well-watered conditions.

#### Effect of CP seed treatment and FC level on seed protein content and protein profiles.

**Fig 5:**
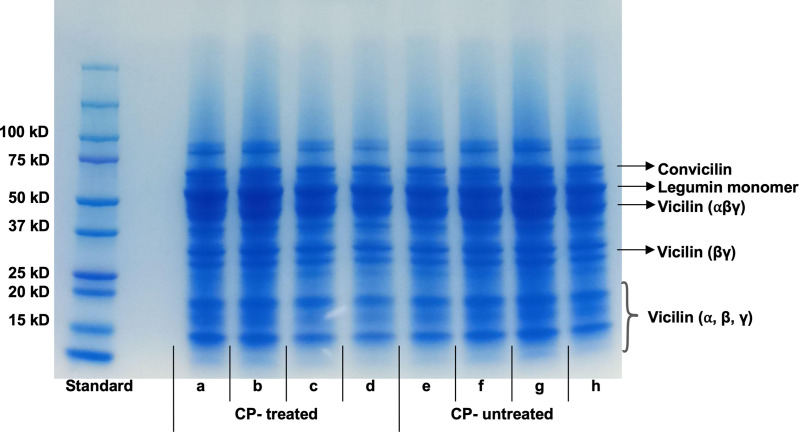
Electrophoretic patterns under non-reducing conditions of pea seed proteins obtained at 80% field capacity. (a), (b), (c), and (d) represent four replicates of CP- treated seeds. (e), (f), (g), and (h) represent four replicates of untreated seeds.

CP seed treatment did not significantly affect seed protein content. However, a slight increasing trend (2%) was observed in seed protein content in CP-treated plants compared to untreated plants (S4 Table). The protein profiles by SDS-PAGE are shown in Fig 5. Bands at 70 kDa, 60 kDa, and 35 kDa corresponded to conviciline, legumin monomers (11S), and viciline [(αβ), categories of globulin protein], respectively. The structure of viciline protein comprises three fractions denoted as α, β, γ, having molecular weights of 19 kDa, 13.5 kDa, and 16 or 12.5 kDa, correspondingly. Therefore, the bands at 35 kDa, 33 kDa and the bands below 20 kDa were attributed to the major and minor subunits of vicilin fractions arranged differently [30]. The protein profiles of untreated and CP-treated seed samples overall were similar, including the bandwidth and intensities. Overall results indicated that CP seed treatment did not significantly alter the seed protein content or the protein profile compared to the untreated control.

## Discussion

Emerging technologies, such as cold plasma, are increasingly important in revolutionizing the agriculture industry by enhancing crop growth, improving yield quantity and quality, and developing resistance to abiotic and biotic stresses while maintaining environmental sustainability. Although cold plasma seed treatment is a less explored technology in crop production, the literature reveals its potential to improve essential crop parameters in legumes. Our studies have identified distinct effects of CP seed treatments on various nodulation parameters, root phenotypic traits, and yield parameters in field pea under different moisture regimes, highlighting its promising role in advancing agricultural practices.

### Impact of drought stress on nodulation, growth, yield, and N fixation in pea

Optimal water availability is essential for plant growth and development and deviations from this optimal soil moisture level can negatively impact plant growth and productivity. Moreover, drought stress diminishes nodulation and SNF capacity [31]. Our experiments also showed that severe to moderate drought stress (30% FC and 45% FC) significantly reduced nodule number (33%-77% reduction) and dry weight per plant (44%-89% reduction) as well as N fixation parameters (seed or shoot total N, %Ndfa, and total fixed N; S2 and S3 Tables). Soil moisture deficit can disrupt the legume-rhizobia signal exchange, resulting in lower nodule production and N fixation [32,33]. Moreover, a reduction in nodule carbon flux due to reduced photosynthesis and sucrose synthase (SS) significantly decreases biological N fixation under drought conditions [34,35]. Additionally, higher production of ethylene under drought stress can reduce nodule numbers as part of the autoregulation of nodulation (AON) system [36].

In our experiments, we observed significant reductions in root morphological parameters (root dry weight, length, surface area, and volume), shoot dry weight, and yield parameters (pod number, pod dry weight, seed number, and seed dry weight) under low moisture regimes (Table 1 and S1 and S2 Tables). The reductions were more pronounced under severe drought conditions (30% FC and 45% FC) and varied with the severity of the moisture drop compared to well-watered conditions. Our findings are in agreement with previous studies using different legumes exposed to drought-stress conditions [37,38]. Severe water shortages can halt plant cell elongation and reductions in leaf area, plant height, root growth, and overall crop growth [31,37,39]. Furthermore, linear electron flow, an indirect measure of photosynthesis, decreased under drought stress conditions in our experiments. Drought stress negatively impacts photosynthesis by disrupting stomatal conductance, electron transport, and the carbon-reduction cycle, leading to lower carbon fixation [40], affecting plant shoot and root growth. Consequently, lower photosynthesis and water scarcity during flowering impair reproductive organs, causing pollen sterility, fewer pods, and lower grain sets [38]. Our results showed a higher SPAD value under drought stress than the well-watered treatment at 50% flowering stage. This rise in leaf chlorophyll content could be associated with a plant adaptation to maintain photosynthesis by enhancing chlorophyll pigments under drought-stress conditions [37,41].

Water use efficiency (WUE), the ratio of fixed carbon to transpired water, is crucial for plant adaptation to drought. Higher WUE is linked to greater drought tolerance and potential productivity [26]. CID is negatively correlated with WUE but positively correlated with yield [42]. Under drought stress, CID decreases due to reduced stomatal function and the water-saving strategies employed by plants. In our experiments, CID was lower at the 30% FC level compared to the 75% FC level at both the 50% flowering and maturity stages, indicating higher water use efficiency under drought stress than well-watered conditions.

### Impact of CP seed treatment on nodulation, growth, yield, and N fixation in pea

Although the seeds treated with CP did not exhibit a significant increase in nodulation parameters (total nodule dry weight, average nodule dry weight, and nodule number per plant) in our current experiments, they showed increasing trends, especially total and average nodule dry weight. This increasing trend in total and average nodule dry weight indirectly suggests that CP seed treatment may enhance N fixation efficiency as the nodule dry weight correlates more strongly with N fixation than the number of nodules [43]. Previous research has shown that treating seeds with cold plasma can significantly enhance legume nodulation [18,20–22,44]. For instance, red clover seedlings from cold plasma-treated seeds exhibited early nodule formation and substantially increased nodule numbers [20]. Our previous study found that seeds treated with cold plasma generated by a DBD system increased the nodule number and nodule dry weight in four weeks old pea plants using the same cultivar we used for the current studies (cv. CDC Meadow) [18]. Specifically, a 6-minute exposure resulted in the highest nodule count per plant (a 136% increase) and the greatest nodule dry weight (a 140% increase) compared to the untreated control [18]. Similarly, soybean plants from CP-treated seeds showed higher average nodule numbers and increased nodule biomass, with elevated nitrogenase activity, leghaemoglobin content, and N content in nodules [22]. Additionally, CP seed treatment influences the release of flavonoids/isoflavonoids from legume roots, facilitating rhizobia attraction and root invasion [20,21,45]. The CP seed treatment also alters phytohormone levels, with higher cytokinin and lower auxin levels in roots, promoting enhanced nodulation phenotypes [22]. However, nodule formation and N fixation are energy-intensive processes regulated by the AON system, which involves hormones, receptor kinases, and small metabolites to control nodule production and rhizobia infection [36]. Although CP seed treatment did not significantly affect %Ndfa, our Experiment 2 results showed increased total N content and fixed nitrogen in seeds, which corresponded to a slight rise in seed protein content. However, SDS-page analysis indicated there is no difference in seed protein functional properties in CP treated and untreated plants. It is important to note that the protein analysis was conducted on only four samples. However, this increase in N content was not observed in the shoots during the 50% flowering stage. The increased nodule number, higher N content, and fixed N content in seeds from cold plasma-treated plants may be due to cold plasma’s direct or indirect influence on specific nodulation phases and plant growth.

In Experiment 1, seed treatment main effect means, and in Experiment 2, CP and FC interaction mean values under 75% FC level, CP seed treatment enhanced root growth (root dry weight, length, surface area, and volume). Recent studies have demonstrated a significant impact of CP seed treatments on legume root growth. For instance, a 5–7 minutes CP exposure significantly increased root length by 27–37% and lateral root numbers by 77–82% in red clover seedlings [20]. Furthermore, CP-treated red clover seedlings exhibited 25% longer roots and 2.5 times higher root weight during the 1–2 trifoliate leaf stage, along with a 50% increase in lateral root and nodule numbers, showing a positive correlation between root size and nodule number [21]. Similar findings in soybean and peanut seedlings indicated that CP treatment enhanced root length and dry weight, regardless of the crop type [19,22,46]. Interestingly, cold plasma-treated soybean seedlings also showed enhanced expression of the *GmEXP1* gene, which promotes root cell elongation through the secretion of expancin, a cell wall protein. Enhanced root length at the 5-day-old stage of soybean was positively correlated with *GmEXP1* expression levels. However, this gene’s expression was decreased in 15-day-old plants, suggesting different developmental phases might influence expansin requirements [22]. These findings indicate that CP seed exposure enhances root architecture, potentially improving water uptake and plant growth.

In Experiment 2, we observed an increase in shoot dry weight with CP seed treatment compared to control plants. Similarly, Experiment 1 showed an upward trend in shoot dry weight in plants from CP seed treatments, indicating the potential of CP seed treatment to enhance plant growth. Although there was no difference in leaf chlorophyll content, plants from CP seed treatments in Experiment 2 exhibited increased LEF, suggesting an improved photosynthetic capacity. Additionally, CP seed-treated plants had higher pod and seed weights than controls, with pod and seed numbers also showing an increasing trend under 75% FC. Previous studies also showed similar results with the CP seed treatment. CP seed-treated peanut plants have shown several growth and productivity improvements, including increased leaf area, thickness, N, and chlorophyll content at the fruiting stage and greater height, stem diameter, and branch number at maturity [19]. These enhancements resulted in a higher pod number, 100-pod weight, and seed yield [19]. Various other studies, including controlled environment and field trials, reported improvements in fresh and dry shoot weight and length in legume plants from CP-treated seeds, such as red clover [20,21,44], soybean [22], and pea [47] during early and later development stages. Moreover, many other crops than legumes also showed improvements in plant growth, photosynthesis, root growth, and productivity with the CP seed treatment [14,44,48]. These findings indicate that CP treatments promoted growth throughout plant development regardless of the genotype.

The CP treatment did not significantly improve root or shoot parameters at 30% FC treatment, although some increasing trends were observed for root parameters. The exact reasons for the underperformance of CP treated seeds under drought stress is unclear. It is possible that elevated levels of ROS played a role. Although ROS serve as crucial signaling molecules in normal plant development, elevated ROS, such as hydrogen peroxide, in the plant tissues can be detrimental causing oxidative damage to lipids, proteins, RNA, DNA, and other small molecules [49]. Abiotic stresses like drought, heat, and salinity is known to increase ROS production, leading to oxidative bursts [49, 50]. CP treatment also contributes to ROS generation [51]. Under severe drought stress, plants produce additional ROS, increasing the likelihood of oxidative damage rather than beneficial signaling. However, previous research showed CP has the ability to increase drought stress resistance in some crops. In a simulated drought study, Adhikari et al. (2020) [52] observed that CP seed-treated tomato plants showed boosted antioxidants, phytohormones, and defense gene expression, improving drought stress tolerance. These distinct patterns can trigger specific signal transduction pathways, activating tailored acclimatization and defense mechanisms under drought stress [50]. It is possible that the underperformance of CP seed treatment under 30% FC in the current study was due to stress-specific ROS signatures and defense mechanisms in pea cv. CDC Meadow but warrants further investigations for confirmation.

## Conclusions

This greenhouse study provides a comprehensive assessment of CP seed treatment effects on yellow field pea performance under drought and well-watered conditions. Our findings indicate that DBD system-generated CP seed treatment for 6 min significantly enhances or shows upward trends in various parameters related to plant growth, photosynthesis, nodulation, and nitrogen fixation under optimal moisture conditions, contributing to higher yield. The enhanced plant growth and yield induced by CP treatment may be due to the direct influence of the CP on altering seed surface properties, germination and root growth, modulating gene expressions related to crop growth and development, or specifically enhancing steps in the SNF process. This leads to a higher N supply to the plants, thereby boosting plant growth and yield under optimal moisture conditions. Under severe drought stress, the beneficial effects of CP treatment were less pronounced, likely due to the oxidative damaged caused by ROS. Despite these limitations, the positive trends observed in some parameters, particularly root parameters, suggest a potential for improved resilience. Further optimization of CP treatment protocols and drought-resistant cultivars could enhance these benefits. Further research is warranted to elucidate the underlying mechanisms and to refine CP treatment strategies with different legume crops, which could pave the way for more sustainable and resilient agricultural practices.

## Supporting information

S1 Table The effect of cold plasma (CP) seed treatment and field capacity (FC) levels on nodulation, root, shoot, and photosynthetic parameters of pea plants at the flowering stage. All parameters were indicated as per plant.
(DOCX)

S2 Table The effect of cold plasma (CP) seed treatment and field capacity (FC) levels on N fixation-related parameters of pea plants at the flowering stage. All parameters were indicated as per plant.
(DOCX)

S3 Table The effect of cold plasma (CP) seed treatment and field capacity (FC) levels on yield parameters of maturity stage pea plants in Experiment 2. All parameters were indicated as per plant.
(DOCX)

S4 Table The effect of CP seed treatment on protein content. All parameters were indicated as per plant.
(DOCX)
