# Supplementary material for: Effects of cold plasma seed treatment on pea (Pisum sativum L.) plant performance under drought and well-watered conditions
Source: PLoS One. 2025 May 2;20(5):e0322108. doi: 10.1371/journal.pone.0322108 (PMC12047786; doi:10.1371/journal.pone.0322108)
Supplement: S1 Table — All parameters were indicated as per plant. (DOCX) [file pone.0322108.s001.docx]

**S1 Table.**  **The effect of cold plasma (CP) seed treatment and field capacity (FC) levels on nodulation, root, shoot, and photosynthetic parameters of pea plants at the flowering stage. All parameters were indicated as per plant.**

| **Treatments** | |  | **Nodule parameters** | | |  | **Root parameters** | | | |  | **Shoot dry weight** |  | **Photosynthetic parameters** | |  |
| --- | --- | --- | --- | --- | --- | --- | --- | --- | --- | --- | --- | --- | --- | --- | --- | --- |
|  |  |  |  |  |  |  |  |  |  |  |  |  |  |  |  |  |
| **Main and interaction effects** | **Seed treatment^⁑^** | **FC^‡^** | **Number of nodules** | **Nodule dry weight (mg)** | **Average nodule dry weight (mg)** |  | **Root dry weight (g)** | **Root length (cm)** | **Root surface area (cm2)** | **Root volume (cm3)** |  | **Shoot dry weight (mg)** |  | **LEF** | **SPAD** |  |
|  | **Control** | **75%** | 102 | 90 | 1.06 |  | 0.222 | 2819 b | 348 b | 3.4 b |  | 3538 |  | 68 | 48 a |  |
|  | **Control** | **30%** | 26 | 13 | 0.50 |  | 0.091 | 1102 c | 121 c | 1.1 c |  | 954 |  | 54 | 58 b |  |
|  | **CP** | **75%** | 103 | 100 | 1.28 |  | 0.260 | 3595 a | 459 a | 4.7 a |  | 4069 |  | 74 | 48 a |  |
|  | **CP** | **30%** | 21 | 9 | 0.46 |  | 0.083 | 1189 c | 135 c | 1.2 c |  | 1022 |  | 55 | 56 b |  |
| **Seed treatment x FC** | |  | **NS^*^** | **NS** | **NS** |  | **NS** | **S** | **S** | **S** |  | **NS** |  | NS | S |  |
|  | **Non-CP** |  | 64 m | 51 m | 0.78 m |  | 0.157 m | 1961 n | 234 n | 2.2 n |  | 2246 n |  | 61 m | 53 m |  |
|  | **CP** |  | 62 m | 54 m | 0.87 m |  | 0.172 m | 2392 m | 297 m | 2.9 m |  | 2545 m |  | 64 m | 52 m |  |
| **Seed treatment** |  |  | **NS** | **NS** | **NS** |  | **NS** | **S** | **S** | **S** |  | **S** |  | NS | NS |  |
|  |  | **75%** | 103 p | 95 p | 1.17 p |  | 0.241 p | 3207 p | 403 p | 4.0 p |  | 3804 p |  | 71 p | 48 q |  |
|  |  | **30%** | 24 q | 11 q | 0.48 q |  | 0.087 q | 1145 q | 128 q | 1.1 q |  | 988 q |  | 54 q | 57 p |  |
| **FC** |  |  | **S** | **S** | **S** |  | **S** | **S** | **S** | **S** |  | **S** |  | S | S |  |

^⁑^Cold plasma (CP) seed treatment was given to pea seeds for 6 mins using a DBD cold plasma generating system. Values in the table are expressed as the mean (n=10).

^‡^FC = Field capacity, pots were maintained at 75% and 30% FC levels.

^*^NS = non-significant, S = significant

^¶^LEF = linear electron flow, ^§^SPAD = leaf chlorophyll content^†^means followed by the same letter indicate means are not significantly different within CP × FC interaction mean values (a-c), among CP treatment main effect means (m, n), and among FC main effect means (p, q) within each parameter by the Tukey’s test, P ≤ 0.05.
