# Supplementary material for: Effects of cold plasma seed treatment on pea (Pisum sativum L.) plant performance under drought and well-watered conditions
Source: PLoS One. 2025 May 2;20(5):e0322108. doi: 10.1371/journal.pone.0322108 (PMC12047786; doi:10.1371/journal.pone.0322108)
Supplement: S2 Table — All parameters were indicated as per plant. (DOCX) [file pone.0322108.s002.docx]

**S2 Table.** **The effect of cold plasma (CP) seed treatment and field capacity (FC) levels on N fixation-related parameters of pea plants at the flowering stage. All parameters were indicated as per plant.**

| **Main and interaction effects** | **Seed treatment^⁑^** | **FC^‡^** | **Shoot total N (mg N)** | **Ndfa%** | **Total shoot N fixed (mg N)** | **CID (^o^/_oo_)** |
| --- | --- | --- | --- | --- | --- | --- |
|  | **Control** | **75%** | 80^†^ | 43.0 | 38.0 | 23 a |
|  | **Control** | **30%** | 32 | 0.3 | 0.3 | 20 b |
|  | **CP** | **75%** | 85 | 41.0 | 35.0 | 23 a |
|  | **CP** | **30%** | 33 | 2.2 | 0.6 | 20 b |
| **Seed treatment x FC** |  |  | NS^⁎^ | NS | NS | S |
|  | **Non-CP** |  | 56 m | 21.7 m | 19.0 m | 22 m |
|  | **CP** |  | 59 m | 21.4 m | 18.0 m | 22 m |
| **Seed treatment** |  |  | NS | NS | NS | NS |
|  |  | **75%** | 83 p | 41.8 p | 36.0 p | 23 p |
|  |  | **30%** | 32 q | 1.2 q | 0.4 q | 20 q |
| **FC** |  |  | S | S | S | S |

^⁑^Cold plasma (CP) seed treatment was given to pea seeds for 6 mins using a DBD cold plasma generating system. Values in the table are expressed as the mean (n=10).

^‡^FC = Field capacity, pots were maintained at 75% and 30% FC levels.

^*^NS = non-significant, S = significant

^†^means followed by the same letter indicate means are not significantly different within CP × FC interaction mean values (a-b), among CP treatment main effect means (m, n), and among FC main effect means (p, q) within each parameter by the Tukey’s test, P ≤ 0.05.
