# Supplementary material for: Effects of cold plasma seed treatment on pea (Pisum sativum L.) plant performance under drought and well-watered conditions
Source: PLoS One. 2025 May 2;20(5):e0322108. doi: 10.1371/journal.pone.0322108 (PMC12047786; doi:10.1371/journal.pone.0322108)
Supplement: S3 Table — All parameters were indicated as per plant. (DOCX) [file pone.0322108.s003.docx]

**S3 Table. The effect of cold plasma (CP) seed treatment and field capacity (FC) levels on yield parameters of maturity stage pea plants in Experiment 2. All parameters were indicated as per plant.**

| **Main and interaction effects** | **Seed treatment^⁑^** | **FC^‡^** | **Pod number** | **Pod weight (g)** | **Seed number** | **Seed weight (g)** |  | **Seed total N (mg N)** | **Ndfa%** | **Total fixed N (mg N)** | **CID (^o^/_oo_)** |
| --- | --- | --- | --- | --- | --- | --- | --- | --- | --- | --- | --- |
|  | **Control** | **75%** | 5.2 a^†^ | 4.5 b | 18 a | 3.7 b |  | 122 b | 84 a | 99 b | 23.4 a |
|  | **Control** | **30%** | 1.9 b | 1.5 c | 6 c | 1.2 c |  | 46 c | 44 b | 21 c | 21.4 c |
|  | **CP** | **75%** | 6.5 a | 6.4 a | 23 a | 5.5 a |  | 206 a | 84 a | 164 a | 23.2 a |
|  | **CP** | **30%** | 1.8 b | 1.1 c | 5 c | 0.9 c |  | 34 c | 50 b | 17 c | 22.2 b |
| **Seed treatment x FC** | |  | NS | S | S | S |  | S | NS | S | S |
|  | **Non-CP** |  | 3.6 m | 3.0 n | 12 n | 2.5 n |  | 84 n | 64 m | 60 n | 22.4 m |
|  | **CP** |  | 4.2 m | 3.8 m | 14 m | 3.2 m |  | 120 m | 68 m | 91 m | 22.7 m |
| **Seed treatment** |  |  | NS | S | NS | S |  | S | NS | S | NS |
|  |  | **75%** | 5.9 p | 5.5 p | 20 p | 4.6 p |  | 164 p | 84 p | 131 p | 23.3 p |
|  |  | **30%** | 1.9 q | 1.3 q | 5 q | 1.1 q |  | 40 q | 47 q | 19 q | 21.8 q |
| **FC** |  |  | S | S | S | S |  | S | S | S | S |

^⁑^Cold plasma (CP) seed treatment was given to pea seeds for 6 mins using a DBD cold plasma generating system. Values in the table are expressed as the mean (n=10).

^‡^FC = Field capacity, pots were maintained at two FC levels 75% and 30%.

^†^means followed by the same letter indicate means are not significantly different within CP × FC interaction mean values (a), among CP treatment main effect means (m), and among FC main effect means (p) within each parameter by the Tukey’s test, P ≤ 0.05.
