# Supplementary material for: Effects of cold plasma seed treatment on pea (Pisum sativum L.) plant performance under drought and well-watered conditions
Source: PLoS One. 2025 May 2;20(5):e0322108. doi: 10.1371/journal.pone.0322108 (PMC12047786; doi:10.1371/journal.pone.0322108)
Supplement: S4 Table — All parameters were indicated as per plant. (DOCX) [file pone.0322108.s004.docx]

**S4 Table. The effect of CP seed treatment on protein content. All parameters were indicated as per plant.**

| **Seed treatment** | **Protein content** |
| --- | --- |
| **CP**^⁑^ | 86.1± 1.5 a^†^ |
| **Control** | 84.5 ± 0.4 a |

^⁑^Cold plasma (CP) seed treatment was given to pea seeds for 6 mins using a DBD cold plasma generating system. Values in the table are expressed as the mean (n=4).

^†^means followed by the same letter indicate means are not significantly different by the two sample T-test, P ≤ 0.05.
